# Associations Between Inflammatory Potential of Diet with the Risk of All-Cause Mortality and Greenhouse Gas Emissions in Chinese Adults

**DOI:** 10.3390/nu17071218

**Published:** 2025-03-30

**Authors:** Zhihan Yao, Yiqian Lv, Wenhui Yang, Man Wu, Shun Li, Huicui Meng

**Affiliations:** 1 School of Public Health (Shenzhen Campus), Sun Yat-Sen University, No. 66, Gongchang Road, Guangming District, Shenzhen 518107, China; 19026072324@163.com (Z.Y.); lvyq7@mail2.sysu.edu.cn (Y.L.); yangwh27@mail2.sysu.edu.cn (W.Y.); wuman5@mail2.sysu.edu.cn (M.W.); listen163123@163.com (S.L.); 2 Guangdong Provincial Key Laboratory of Food, Nutrition and Health, Guangzhou 510080, China; 3 Guangdong Province Engineering Laboratory for Nutrition Translation, Guangzhou 510080, China

**Keywords:** dietary inflammatory index, dietary inflammatory potential, pro-inflammatory diet, all-cause mortality, greenhouse gas emission, Chinese adults

## Abstract

**Background:** Current research inadequately substantiates the impacts of dietary inflammatory potential based on the dietary inflammatory index (DII) on population health and environmental sustainability in Chinese adults. **Objectives:** We aimed to investigate the associations between the DII with the risk of all-cause mortality and greenhouse gas (GHG) emissions in Chinese adults. **Methods:** Data from adults (N = 15,318) in the China Health and Nutrition Survey 1997–2015 wave were included in the analysis. DII and energy-adjusted DII (E-DII) were calculated using dietary intake data collected with a combination of 3-day consecutive 24-h dietary recalls and the food weighing method. The total GHG emissions were calculated by summing the amount of emissions from all the food groups consumed by the participants. Cox proportional hazards regression models and linear regression models were conducted for statistical analysis. **Results:** A pro-inflammatory diet, as reflected by higher DII and E-DII scores was associated with an increased risk of all-cause mortality (DII: Q5 vs. Q1: HR = 1.82; 95% CI: 1.45–2.30; *p*-trend < 0.0001; E-DII: Q5 vs. Q1: HR = 1.86; 95% CI: 1.38–2.52; *p*-trend < 0.0001) and higher amounts of GHG emissions (both *p*-trend < 0.0001). **Conclusions:** These findings demonstrated positive associations between pro-inflammatory potentials with an increased risk of all-cause mortality and higher GHG emissions among Chinese adults, suggesting dual adverse impacts of a pro-inflammatory diet on health and environmental sustainability.

## 1. Introduction

With the widespread prevalence of unhealthy lifestyles such as an unhealthy diet and lack of physical activity, the mortality of chronic diseases including cardiometabolic diseases and cancer has been rising annually worldwide over the past 30 years, posing a significant public health challenge [1,2]. According to the Global Burden of Disease Study, an unhealthy diet is one of the main risk factors for premature death and disability-adjusted life years (DALYs) among adults [3].

Inflammation is the immune response of the body to inflammatory stimuli or cellular damage [4], which is a normal physiological reaction. However, if the inflammatory response persists, it can lead to chronic low-grade inflammation, which is significantly associated with a risk of cardiometabolic diseases, cancer, and premature deaths in adults [4,5,6]. Numerous studies have found that chronic low-grade inflammation is closely related to mortality [7,8]. Furthermore, long-term chronic low-grade inflammation can lead to inflammaging, where chronic inflammation accelerates the aging of immune cells, resulting in a weakened immune function. This impairs the body’s ability to clear senescent cells and inflammatory factors, creating a vicious cycle of inflammation and aging [9], thereby being associated with mortality.

Previous studies have reported associations between several nutrients, foods, and dietary patterns with chronic low-grade inflammation [10,11,12,13]. Diet can influence inflammation-related diseases and premature death via modulating the chronic inflammation status [14,15,16,17,18,19]. Several indices have been proposed to reflect the inflammatory potential of diet and assess its relationship with health and disease outcomes. Although there is currently no standardized index to measure the inflammatory potential of a diet, the dietary inflammatory index (DII) and energy-adjusted dietary inflammation index (E-DII) have been established on the basis of a wide array of the literature and validated against six inflammatory biomarkers. Positive associations between the DII and risk of cardiometabolic diseases and cancer have been reported in diverse populations [20,21,22,23,24,25]. Several prospective cohort studies [26,27,28,29,30] and meta-analyses [31,32,33,34,35] have reported positive associations between the DII and risk of all-cause mortality. The associations between the DII and risk of all-cause mortality among Chinese adults have been previously investigated in two prospective cohort studies. A prospective cohort study with a mean follow-up time of 5 years has reported positive associations between the DII and the risk of all-cause and cause-specific mortality among adults in two rural towns of Xin’an County, Luoyang, China [36]. Another prospective cohort study, based on data from the China Health and Nutrition Survey (CHNS) from wave 2004 to 2011, has reported no significant association between the E-DII and risk of all-cause mortality among Chinese adults [37]. Furthermore, discrepancies have been observed in earlier studies regarding the relationship between the DII and risk of all-cause mortality among individuals with different age, sex, body mass index (BMI), and health status, which are factors linked to the variability in the inflammatory status [36,38]. Updated data from more representative populations are required to explore the relationships between the dietary inflammatory potential, as reflected by the DII and E-DII, and the risk of all-cause mortality among Chinese adults.

Diet, population health, and planetary health are closely interconnected. Based on previous research, activities within food systems, such as food production, food processing, and waste disposal, account for almost 30% of global greenhouse gas (GHG) emissions [39,40,41]. Consequently, diets and food systems have been identified as one of the major contributors to climate change, which in turn impacts the quantity, quality, diversity, and safety of foods and diets [40,41,42]. Moreover, foods with a higher degree of processing could lead to greater GHG emissions [43]. Transitioning to sustainable diets can provide long-term benefits for both planetary and population health. Our previous study has reported inverse associations between the adherence to healthy and sustainable plant-based dietary patterns with higher DII scores, as well as positive associations between the adherence to unhealthy plant-based dietary patterns with higher DII scores [24]. Healthy and sustainable plant-based dietary patterns are linked to low environmental impacts, such as low GHG emissions, which pose significant challenges to global planetary and population health, notably by promoting the development of cardiometabolic diseases and premature adult death worldwide [44]. There is currently no evidence from prospective cohort studies regarding the associations between diets with higher inflammatory potentials and GHG emissions, and further investigations are required.

The current study aimed to investigate the associations between the inflammatory potential of diets, as reflected by the DII and E-DII, with the risk of all-cause mortality in a nation-wide prospective cohort of Chinese adults. It also sought to assess the environmental impacts of a diet with a higher inflammatory potential using GHG emissions within the same cohort. We hypothesized that higher DII and E-DII scores, indicative of a pro-inflammatory diet, would be associated with a higher risk of all-cause mortality as well as GHG emissions in Chinese adults.

## 2. Methods

### 2.1. Study Population

Data of the current study were obtained from CHNS, which is an ongoing longitudinal open cohort study aimed at assessing the impacts of policy implementation and socioeconomic transformation on the health and nutritional status of Chinese populations. The CHNS cohort study employs a multistage random cluster sampling process and completed 10 waves of surveys. It recruited approximately over 30,000 individuals from 7200 households in 15 provinces, cities, and autonomous regions from 1989 to 2015. Detailed information of CHNS has been provided previously [45,46]. All the survey protocols were approved by the Institutional Review Board of the University of North Carolina at Chapel Hill and the National Institute for Nutrition and Health at the Chinese Center for Disease Control and Prevention [47,48]. Written informed consent was obtained from all the participants prior to their participation in the survey [46].

**Figure 1:**
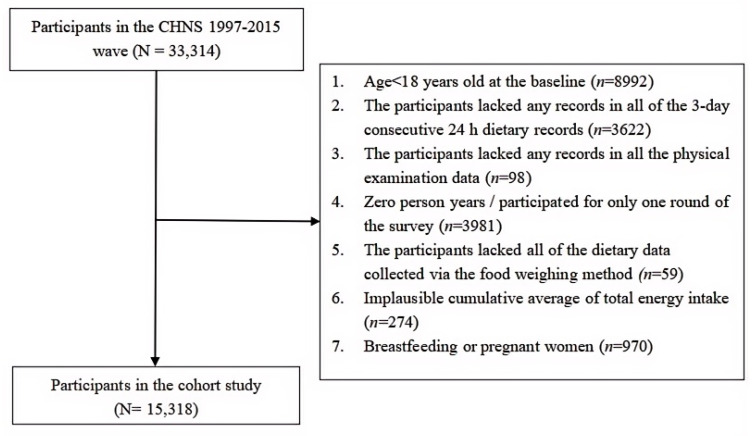
Screening and excluding process for participants in the cohort study in the China Health and Nutrition Survey 1997–2015 wave.

This prospective cohort study utilized data from the CHNS 1997–2015 wave to investigate the associations between the inflammatory potential of diets, as reflected by the DII and E-DII, and the risk of all-cause mortality. Initially, 33,314 participants were selected in the cohort. The baseline of the participants was defined as the date of their initial dietary intake survey during the 1997–2015 wave. Participants were excluded if they were under 18 years old at the baseline (*n* = 8992), lacked any records from the 3-day consecutive 24-h dietary recalls (*n* = 3622), had no physical examination data (*n* = 98), participated in only one wave of the survey (*n* = 3981), or had no dietary data collected via the food weighing method (*n* = 59). Participants were further excluded if they had an implausible cumulative average of total energy intake (<800 or >4200 kcal/day for males; <500 or >3500 kcal/day for females, *n* = 274) [49], or were pregnant or breastfeeding women (*n* = 970). Ultimately, 15,318 participants were included in the final analysis, including 7760 males and 7558 females (Figure 1).

### 2.2. Dietary Intake Assessment and Calculation of the DII and E-DII

In the current study, the dietary intake data of the participants were collected using a combination of 3-day consecutive 24-h dietary recalls at the individual level and the food weighing method at the household level. These methods have been validated in previous studies for assessing food and nutrient intakes in the CHNS cohort [50,51]. Trained interviewers collected detailed information on the type, amount, preparation methods, and dining location of all the foods and beverages consumed by the participants at home and away from home over three consecutive days, including two weekdays and one weekend. The consumption of cooking oils and condiments was determined using a food weighing method at the household level [52]. All the data collections were conducted by trained interviewers to ensure accuracy and consistency. Additionally, dietary intake data at the individual level were compared and cross-checked with household food intake data over the same three days for validation.

The daily average amounts of food and nutrient intakes over three days were then calculated by referencing the edible portion and nutrient contents of food items in the Chinese Food Composition Table [53,54,55]. Dietary records were updated at each survey wave, and a total of six rounds were conducted during the 1997 to 2011 wave. In order to represent long-term dietary habits and reduce intra-individual variability, the cumulative average of daily food and nutrient intakes from the baseline to death was used in the analysis [56,57].

The calculation of the DII has been described in detail previously [24]. Briefly, following the method developed by Shivappa et al. [20], a total of 29 food parameters are involved in the calculation of the DII. The Z-score for each dietary component was calculated based on daily average intakes, standardized by reference to global daily average intakes, converted into centered percentile values ranging from −1 to 1, and multiplied by the specific inflammatory effect score of each food parameter to obtain the DII score for that food parameter. Finally, the DII scores of the 29 dietary components were summed to obtain the total DII score for each participant.

The E-DII was calculated based on the intake of dietary components per 1000 kcal, which utilized the energy-standardized version of the world database, serving as an indicator to control for the influence of total energy intake [58]. A relatively higher DII or E-DII score (especially a score greater than 1) indicates a diet with a pro-inflammatory potential, while a relatively lower DII or E-DII score (especially a score lower than −1) indicates a diet with an anti-inflammatory potential [16,58].

### 2.3. Assessment of GHG Emissions

GHG emissions associated with the entire cycle of food production were estimated following a previously established method [59] to assess the environmental impact of a pro-inflammatory diet. Concisely, GHG emissions per gram of various types of foods and food groups were referenced from a previous study, which collected data over 100 life cycle assessment studies, covering the entire production process up to the farm gate [59]. The GHG emissions of each food or food group were calculated by multiplying the average emissions per gram of food or food group in the unit of grams of CO_2_ equivalents (gCO_2e_), as determined from the CHNS dataset, by the quantity of corresponding food or food group consumed by each individual. Finally, the total GHG emissions of each participant were obtained by summing the GHG emissions of all foods and food groups consumed by this participant, and the results were expressed in the unit of gCO_2e_. The cumulative average of amounts of GHG emissions from the baseline to death was used in the analysis.

### 2.4. Ascertainment of Death

In this study, the date of the participants’ initial enrollment during 1997–2015 was utilized to define the baseline, and the date of occurrence of death during the follow-up time was utilized as the outcome time. The death status and exact date of death were determined based on information from family members in each survey year [60]. In the case of conflicts in the records of the death status, the earliest reported date of death was used [37,61]. In the case of unavailability of the dietary survey date, priority was given to the date of questionnaires from the same wave. The follow-up time of each participant was calculated from the baseline until death, or until the last wave before exiting the survey, or until the end of wave 2015, whichever occurred first [62].

### 2.5. Assessment of Covariates

The information collection on sociodemographic and lifestyle characteristics and anthropometric measurements of the participants were conducted using the established methods as described previously [24].

### 2.6. Statistical Analysis

All the statistical analyses were performed using SAS 9.4 software (SAS Institute, Cary, NC, USA). Baseline sociodemographic and lifestyle characteristics, anthropometric measurements, and the dietary intakes of the participants were presented based on the quintiles of the baseline DII and E-DII scores. Continuous variables were presented as mean ± standard deviation (SD) or median (P_25_, P_75_) and categorical variables were presented as N (%). The dietary intake data across the quintiles of the DII and E-DII groups were compared using the independent-sample Kruskal–Wallis rank-sum test.

Cox proportional hazard regression models were used to assess the associations between the inflammatory potential of diet, as reflected by the DII and E-DII scores, and the risk of all-cause mortality. Cumulative average values of dietary intake, BMI, urbanization index, and physical activity data from the baseline to the date of death or the end of follow-up were used in the analysis to represent long-term dietary habits and reduce intra-individual variations [56]. The independent variables were quintiles of the DII and E-DII scores, and the lowest quintile of each index was used as the reference to estimate the hazard ratio (HR) and 95% confidence intervals (95% CI) for the risk of all-cause mortality in the Cox regression analysis. Both indices were fitted in separate Cox models. In the analysis with the DII, model 1 adjusted for age (<50 years old, 50–54 years old, 55–59 years old, 60–64 years old, and ≥65 years old) and sex (male or female). Model 2 additionally adjusted for other confounders, including the BMI [underweight (<18.5 kg/m^2^), normal weight (18.5–23.9 kg/m^2^), overweight (24–27.9 kg/m^2^), or obese (≥28 kg/m^2^)], education level (elementary, middle, or high), region (north or south), urbanization index [low (23.0–50.0), medium (50.0–75.0), or high (75.0–103.0)], physical activity (low, medium, or high), baseline hypertension [no (SBP < 140 mmHg and DBP < 90 mmHg) or yes (SBP ≥ 140 mmHg or DBP ≥ 90 mmHg)], smoking status (yes or no), drinking status (yes or no), and total energy intake (quintiles). Model 1 for the E-DII was the same as for the DII, and model 2 for the E-DII did not adjust for the total energy intake. A test for linear trends was performed with the DII and E-DII scores as continuous variables by assigning the median values of Q1-Q5 of these indices to the variables in the Cox regression model. Additionally, continuous variables of the DII and E-DII scores were standardized to Z-scores, and the per SD increases in the DII and E-DII scores were used as the independent variables in the Cox regression analyses to estimate the HR and 95% CI for the risk of all-cause mortality. Confounders were consistent with models 1 and 2 of the Cox regression models with the quintiles of the DII and E-DII scores.

Linear regression models were utilized to estimate the Least Squares Means (95% confidence level, 95% CL) of the amounts of GHG emissions to investigate the associations between the DII and E-DII scores and the amount of GHG emissions. The same confounding factors as in the Cox regression models were adjusted for in models 1 and 2 of the linear regression analysis.

Stratified analysis and potential effect modification were tested for the associations between the DII and E-DII scores and risk of all-cause mortality by age (<60 or ≥60 years old), sex (male or female), BMI (<24 or ≥24 kg/m^2^), region (northern or southern), baseline hypertension (yes or no), and food-derived GHG emissions [lower than the median (<4861.18 gCO_2e_) or higher than the median (≥4861.18 gCO_2e_)]. Confounders were consistent with model 2 of the Cox regression models, and stratification variables were not included in the corresponding models.

Two types of sensitivity analyses were conducted to test the stability of the results. The first analysis was conducted by excluding those participants who had myocardial infarction, type 2 diabetes, stroke, or cancer, or took medicines to treat these diseases at the baseline. The second analysis was conducted by excluding those participants who died within the first two years of follow-up.

A restricted cubic spline (RCS; SAS macro program %RCS_Reg) Cox proportional hazard regression model with 3 knots (10th, 50th, and 90th) was performed to test the potential non-linear and dose-response relationships between the DII, E-DII, and risk of all-cause mortality [63]. Confounders were consistent with model 2 of the Cox regression models. The median values of the DII and E-DII were used as a reference in the RCS analysis. All the tests were two-tailed, and the differences were considered statistically significant when *p* < 0.05.

## 3. Results

### 3.1. Sociodemographic, Anthropometric, and Lifestyle Characteristics and Dietary Intakes of the Study Participants at the Baseline

A total of 15,318 participants were included in the prospective cohort study, and 1343 cases of deaths (154,485 person-years) were identified after a mean follow-up of 10 years. At the baseline, the median (P_25_, P_75_) of the DII scores was −0.01 (−1.01, 1.00), with a range from −4.45 to 4.04, and the median (P_25_, P_75_) of the E-DII scores was −0.01 (−0.88, 0.88), with a range from −4.15 to 4.65. Compared to the participants with lower DII and E-DII levels (anti-inflammatory diet), those with higher levels (pro-inflammatory diet) were more likely to have low education levels, and live in the southern regions of China and in areas with medium urbanization (Table 1).

Compared to the participants with lower DII and E-DII scores (anti-inflammatory diet), those with higher scores (pro-inflammatory diet) were more likely to have higher intakes of total fat, saturated fatty acid (SFA), and monounsaturated fatty acid (MUFA), and lower intakes of other nutrients, except for higher intakes of total energy, cholesterol and sodium in the participants with higher compared to lower E-DII scores (Table 2). Additionally, the participants with higher DII and E-DII scores consumed lower amounts of whole grains, fruits, vegetables, nuts, legumes, dairy products, and eggs in comparison to those with lower scores (all *p* < 0.05) (Table 2). The participants with higher E-DII scores also had higher intakes of red and processed meat in comparison to those with lower scores (*p* < 0.0001).

### 3.2. Associations Between the DII, E-DII, and Risk of All-Cause Mortality

In the models adjusted for age and sex, both the DII and E-DII were associated with an increased risk of all-cause mortality (model 1; both *p*-trend < 0.0001) (Table 3). In the fully adjusted models, the participants with higher DII and E-DII scores (pro-inflammatory diet) had an 82% (model 2; Q5 vs. Q1: HR = 1.82; 95% CI: 1.45–2.30; *p*-trend < 0.0001) and 86% (model 2; Q5 vs. Q1: HR = 1.86; 95% CI: 1.38–2.52; *p*-trend < 0.0001) higher risk of all-cause mortality compared to those with lower DII and E-DII scores (anti-inflammatory diet), respectively (Table 3). Additionally, every 1 SD increase in the DII and E-DII scores was associated with a 25% (model 2; per SD increase: HR = 1.25; 95% CI: 1.16–1.35; *p* < 0.0001) and 27% (model 2; per SD increase: HR = 1.27; 95% CI: 1.15–1.41; *p* < 0.0001) increased risk of all-cause mortality (Table 3).

### 3.3. Associations Between the DII, E-DII, and GHG Emissions

**Figure 2:**
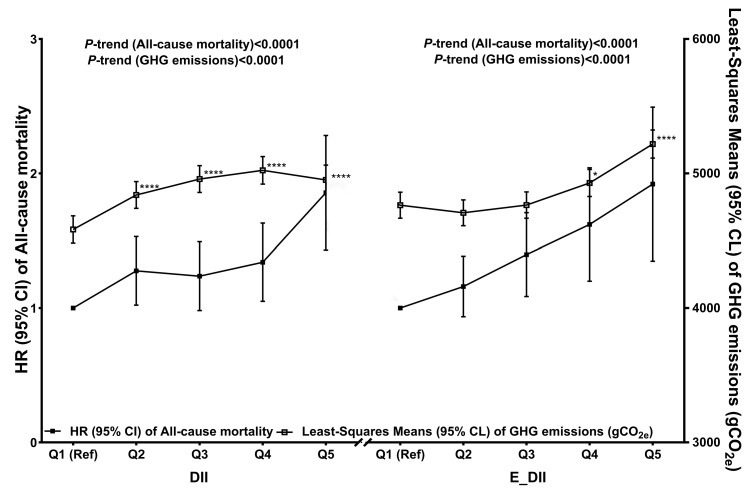
Associations between pro-inflammatory diet indices with GHG emissions and risk of all-cause mortality (N = 15,318) in Chinese adults who participated in the China Health and Nutrition Survey 1997–2015 wave. The corresponding numerical data are listed in Table 3 and Table 4. Data for all-cause mortality (solid squares) are presented as HR (95% CI) estimated via Cox proportional hazard regression models. Model for DII adjusted for sex and age, BMI, region, urbanization index, educational level, physical activity, baseline hypertension, smoking status, alcohol intake, and total energy intake. Model for E-DII adjusted for sex and age, BMI, region, urbanization index, educational level, physical activity, baseline hypertension, smoking status, alcohol intake, and total energy intake. Data for GHG emissions (hollow squares) are presented as Least Squares Means (95% CL) estimated by using multiple linear regression models, and models adjusted for same confounding factors as in Cox proportional hazard regression models. Abbreviations: CI, confidence interval; CL, confidence level; gCO_2e_, grams of CO_2_ equivalents; GHG, greenhouse gas; HR, hazard ratio; Q, quintile; Ref, reference; DII, dietary inflammatory index; E-DII, energy-adjusted dietary inflammation index. * Compared with Q1 (GHG emissions), *p* < 0.05; **** compared with Q1 (GHG emissions), *p* < 0.0001.

The Least Squares Means of GHG emissions by reference to the quintiles of the DII and E-DII in the study population are shown in Table 4. In both models, GHG emissions significantly increased across the quintiles of the DII and E-DII scores, with a slight decrease observed in Q5 for the DII model (all *p*-trends < 0.0001) (Table 4 and Figure 2). Compared with the participants in the lowest quintiles of the DII and E-DII scores, the participants in the highest quintiles had 8.03% to 9.57% higher amounts of GHG emissions (model 2; 8.03%: Q5 of the DII 4950.56 vs. Q1 of DII 4582.44; model 2; 9.57%: Q5 of the E-DII 5218.12 vs. Q1 of E-DII 4706.90) (Table 4 and Figure 2).

### 3.4. Associations Between the DII, E-DII, and Risk of All-Cause Mortality on the Basis of Potential Effect Modifiers

**Figure 3:**
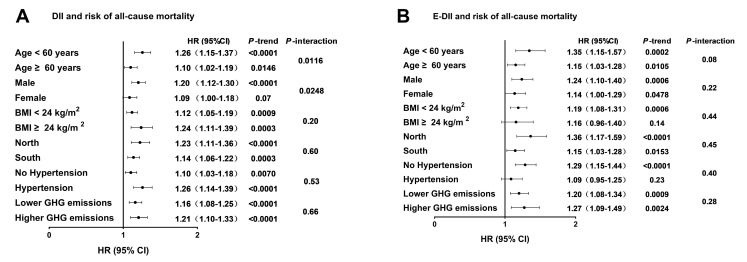
Associations between DII (**A**), E-DII (**B**) and risk of all-cause mortality in 15,318 Chinese adults who participated in the China Health and Nutrition Survey 1997–2015 wave, stratified by age, sex, BMI, region, baseline hypertension history, and GHG emissions. Data are presented as HR (95% CI) for quintile 5 compared to quintile 1 (reference) estimated by using Cox proportional hazard regression models. Models for DII adjusted for age, sex, BMI, education level, urbanization index, region, smoking status, alcohol consumption, physical activity status, baseline hypertension, and total energy intake. Models for E-DII adjusted for age, sex, BMI, educational level, urbanization index, region, smoking status, alcohol consumption, physical activity status, and baseline hypertension. In the analyses of interactions between the DII, E-DII, and GHG emissions on all-cause mortality, models additionally adjusted for GHG emissions. Abbreviations: DII, dietary inflammatory index; E-DII, energy-adjusted dietary inflammation index; GHG emissions, greenhouse gas emissions.

Age (*p*-interaction = 0.0116) and sex (*p*-interaction = 0.0248) significantly modified the associations between the DII and risk of all-cause mortality. The positive association between the DII and risk of all-cause mortality was stronger in the young and middle-aged participants (<60 years old: Q5 vs. Q1: HR = 1.26; 95% CI: 1.15–1.37; *p*-trend < 0.0001) compared to the older participants (≥60 years old: Q5 vs. Q1: HR = 1.10; 95% CI: 1.02–1.19; *p*-trend = 0.0146). There was a positive association between the DII and risk of all-cause mortality in the male participants (Q5 vs. Q1: HR = 1.20; 95% CI: 1.12–1.30; *p*-trend < 0.0001). However, no significant association between the DII and risk of all-cause mortality was observed in the female participants (Q5 vs. Q1: HR = 1.09; 95% CI: 1.00–1.18; *p*-trend = 0.07). There was no evidence of effect modifications of the associations between the DII and risk of all-cause mortality due to the BMI, region, or baseline hypertension, and between the E-DII and risk of all-cause mortality due to any of the effect modifiers (all *p*-interaction > 0.05). There was also no evidence of effect modifications of the associations between the DII and E-DII with risk of all-cause mortality due to food-derived GHG emissions (both *p*-interaction > 0.05). However, regardless of the lower or higher food-derived GHG emissions, the DII and E-DII were positively associated with the risk of all-cause mortality (all *p*-trend < 0.05) (Figure 3).

The sensitivity analyses excluding cases of deaths within two years of follow-up and those participants who had myocardial infarction, type 2 diabetes, stroke, or cancer, or took medicines to treat these diseases at the baseline demonstrated similar results to those of the main analyses (Supplementary Table S1).

### 3.5. The Nonlinear and Dose–Response Relationships Between the DII, E-DII, and Risk of All-Cause Mortality

**Figure 4:**
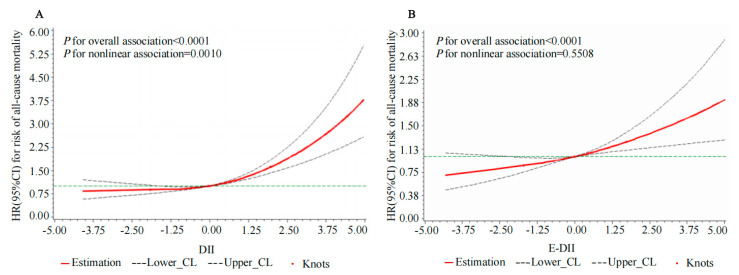
Non-linear associations between DII (**A**), E-DII (**B**), and risk of all-cause mortality in 15,318 Chinese adults who participated in the China Health and Nutrition Survey 1997–2015 wave. Data are estimated by using restricted cubic spline Cox proportional hazard regression model. Model for DII adjusted for age, sex, BMI, educational level, urbanization index, region, smoking status, alcohol consumption, physical activity status, baseline hypertension, and total energy intake. Model for E-DII adjusted for age, sex, BMI, educational level, urbanization index, region, smoking status, alcohol consumption, physical activity status, and baseline hypertension. The green dashed line represented reference. Abbreviations: DII, dietary inflammatory index; E-DII, energy-adjusted dietary inflammation index.

RCS analysis showed a nonlinear association between the DII and risk of all-cause mortality (*p* for overall association < 0.0001, *p* for nonlinear association = 0.0010) (Figure 4). The DII was associated with an increased risk of all-cause mortality when the DII score exceeded −0.04 (Figure 4). There was no significant nonlinear relationship between the E-DII and risk of all-cause mortality (*p* for overall association < 0.0001, *p* for nonlinear association = 0.55) (Figure 4).

## 4. Discussion

In accordance with our hypothesis, the current study demonstrated long-term positive associations between a pro-inflammatory diet, as represented by the higher DII and E-DII scores, and increased risk of all-cause mortality. These associations remained significant even after adjusting for a wide array of confounders and in sensitivity analyses. In terms of environmental sustainability, higher DII and E-DII scores were associated with higher GHG emissions. To the best of our knowledge, the current study provides the first documentation of associations between the inflammatory potential of diet, as assessed via the DII and E-DII, and GHG emissions, highlighting the adverse impact of a pro-inflammatory diet on both health and environmental sustainability.

A pro-inflammatory diet, as reflected by a higher DII or E-DII score, was associated with a higher risk of all-cause mortality, which was consistent with our hypothesis and a previous study in Chinese adults [36]. These findings also align with studies conducted in other populations [26,27,28,29,30]. In the current and our previous study [24], in comparison to participants with lower DII and E-DII scores, those with higher DII and E-DII scores had significantly higher intakes of pro-inflammatory food components, including total fat and SFA, while the intakes of anti-inflammatory food components were lower, including whole grains, fruits, vegetables, nuts, legumes, dietary fiber as well as antioxidant vitamins and minerals. The higher intakes of pro-inflammatory food components and lower intakes of anti-inflammatory food components collectively contribute to higher pro-inflammatory potentials of diet, which are associated with higher chronic low-grade inflammation as demonstrated by elevated high-sensitivity C-reactive protein concentrations [21,24,27,64]. The potential mechanisms underlying the connections between a pro-inflammatory diet and chronic low-grade inflammation may include the promotion of innate immune responses and release of pro-inflammatory biomarkers via diet-related endotoxins [65], the dysregulation of glucose homeostasis [66,67], and endothelial dysfunction [68] as consequences of long-term adherence to a pro-inflammatory diet. A pro-inflammatory diet may also contribute to inflammaging either directly via elevations in chronic low-grade inflammation [69] or indirectly via alterations in diet-induced changes in the gut microbiota and gut-derived metabolites [70]. Chronic low-grade inflammation and inflammaging are important risk factors for a variety of aging-related chronic diseases and the related mortality [6,24,35,71,72]. Of note, we and others have reported associations between the pro-inflammatory potentials of diet with a lower adherence to healthy and sustainable plant-based dietary patterns, such as the EAT–Lancet reference diet and the Mediterranean diet [24,73,74,75]. These healthy and high-quality dietary patterns have been extensively documented to be related to a reduced risk of all-cause mortality, which may also contribute to the positive associations between the pro-inflammatory potentials of diet and risk of all-cause mortality [76,77,78,79].

This study also, for the first time, identified a significant positive association between a pro-inflammatory diet, as reflected by higher DII and E-DII scores, and GHG emissions. These findings indicate dual adverse impacts of a pro-inflammatory diet on both population and environmental health. Animal-based foods, particularly red and processed meat, have a significantly higher carbon footprint and GHG emissions than plant-based foods due to the resource-intensive nature of animal farming [80]. In addition, according to the EAT–Lancet Commission [81], transitioning towards plant-based diets, which tend to have lower DII and E-DII scores [24], could reduce global GHG emissions by more than 40%, while simultaneously lowering the risk and mortality of chronic diseases. Collectively, the adherence to healthy plant-based dietary patterns with lower inflammatory potentials may have dual benefits in both health and environmental sustainability.

Age and sex significantly modified the associations between the DII and the risk of all-cause mortality. Young and middle-aged adults had a stronger association between high DII scores and increased mortality risk compared to older adults. This may be attributed to the higher mortality rates in older adults [82] and confounding factors such as aging-related injuries, which may weaken the association between the DII and risk of all-cause mortality [83]. The positive association between the DII and risk of all-cause mortality was only observed in the male participants. The estrogens and X chromosomes of females were associated with lower rates of infection and chronic inflammatory diseases [84]. Chinese women tend to have a greater dietary diversity and healthier dietary patterns, whereas men have a greater preference for high-fat or high-salt dietary patterns [85]. These reasons collectively may contribute to the lesser impact of a pro-inflammatory diet on females than males, resulting in sex differences in the association between the DII and mortality risk.

This study has several strengths. It utilized data from CHNS, which is meticulously designed and conducted. The survey employs a multi-stage, random cluster process, with systematic and professional training for interviewers and rigorous quality control measures. The survey covers approximately 7200 households and over 30,000 individuals across 15 provinces and municipalities, which are highly representative of the Chinese population. The DII and E-DII were used to assess the dietary inflammatory potentials, which have been validated in numerous previous studies.

The current study also has several limitations. The calculations of the DII and E-DII in this study were based on 29 food parameters, which were fewer than the 45 food parameters proposed in the initial development of the DII and E-DII [20]. Some important pro-inflammatory or anti-inflammatory food parameters may be overlooked by using a smaller number of food parameters to calculate the DII and E-DII. However, previous studies reported an average of 27 food parameters used in the DII or E-DII calculations [38], and the food parameters that were not used in the current study were foods or condiments rarely consumed by Chinese adults. Due to the lack of data on cause-specific mortality, the analysis was only conducted with all-cause mortality in this study. This study employed a prospective cohort study design, and the causality between dietary inflammatory potentials and all-cause mortality risk could not be established due to the observational nature. The data of GHG emissions and dietary intakes were collected and analyzed simultaneously, so the sequential causal relationship between them needs to be further clarified. Among the environmental parameters, only GHG emission was analyzed, which could not be representative of environmental sustainability. Although a wide array of potential confounders was adjusted in the analysis, we could not exclude the possibility of other confounders.

## 5. Conclusions

In summary, the current study demonstrated long-term positive associations between the adherence to a pro-inflammatory diet, characterized by higher DII and E-DII scores, and an increased risk of all-cause mortality among Chinese adults. We also, for the first time, reported associations between both DII and E-DII scores with greater amounts of GHG emissions. These findings collectively suggest the potential dual adverse impacts of a pro-inflammatory diet on health and environmental sustainability. Further research is needed to explore the causal relationships and develop food-based dietary recommendations aimed at improving both population health and environmental sustainability.

## Figures and Tables

**Table 1:** Baseline sociodemographic, anthropometric, and lifestyle characteristics of 15,318 Chinese adults who participated in the China Health and Nutrition Survey 1997–2015 wave based on quintiles of DII and E-DII.

Variables	Total	Quintiles of DII			Quintiles of E-DII		
		Q1	Q3	Q5	Q1	Q3	Q5
N	15,318	3063	3063	3063	3064	3064	3063
DII	−0.01 (−1.01, 1.00)	−1.84 (−2.25, −1.51)	−0.01 (−0.21, 0.19)	1.85 (1.51, 2.31)	−1.22 (−2.00, −0.25)	−0.09 (−0.81, 0.75)	1.23 (0.46, 2.01)
E-DII	−0.01 (−0.88, 0.88)	−1.08 (−1.80, −0.44)	−0.08 (−0.63, 0.74)	1.10 (0.24, 1.87)	−1.69 (−2.16, −1.35)	−0.01 (−0.18, 0.16)	1.67 (1.36, 2.14)
Age, years	46 ± 15	44 ± 14	45 ± 15	49 ± 17	48 ± 15	45 ± 15	45 ± 15
Male, n (%)	7760 (50.7)	1911 (62.4)	1529 (49.9)	1200 (39.2)	1332 (43.5)	1581 (51.6)	1688 (55.1)
BMI, kg/m^2^	22.6 (20.6, 25.1)	22.7 (20.7, 25.2)	22.7 (20.6, 25.1)	22.4 (20.4, 24.8)	23.2 (21.0, 25.6)	22.6 (20.6, 25.0)	22.1 (20.2, 24.5)
SBP, mm Hg	120.0 (110.0, 130.0)	120.0 (110.0, 130.0)	120.0 (110.0, 130.0)	120.0 (110.0, 131.7)	120.0 (110.0, 131.3)	120.0 (110.0, 130.0)	119.3 (108.7, 129.0)
DBP, mm Hg	79.3 (70.0, 84.0)	80.0 (70.0, 84.7)	78.7 (70.0, 83.3)	78.7 (70.0, 83.3)	79.7 (70.0, 85.0)	79.3 (70.0, 83.3)	77.3 (70.0, 82.7)
Education level, n (%)							
Primary	7186 (46.9)	1401 (45.7)	1398 (45.6)	1569 (51.2)	1252 (40.9)	1463 (47.7)	1492 (51.2)
Middle	4353 (28.4)	925 (30.2)	883 (28.8)	783 (25.6)	844 (27.5)	898 (29.3)	892 (29.1)
High	3779 (24.7)	737 (24.1)	782 (25.5)	711 (23.2)	968 (31.6)	703 (22.9)	679 (22.2)
Urbanization index, n (%)							
Low	5085 (33.2)	1243 (40.6)	993 (32.4)	845 (27.6)	785 (25.6)	1154 (37.7)	982 (32.1)
Medium	5089 (33.2)	817 (26.7)	1042 (34.0)	1173 (38.3)	793 (25.9)	956 (31.2)	1294 (42.2)
High	5144 (33.6)	1003 (32.7)	1028 (33.6)	1045 (34.1)	1486 (48.5)	954 (31.1)	787 (25.7)
Region, n (%)							
Northern	6400 (41.8)	1465 (47.8)	1198 (39.1)	1073 (35.0)	1551 (50.6)	1398 (45.6)	799 (26.1)
Southern	8918 (58.2)	1598 (52.2)	1865 (60.9)	1990 (65.0)	1513 (49.4)	1666 (54.4)	2264 (73.9)
Current smokers, n (%)	4813 (31.4)	1156 (37.7)	940 (30.7)	754 (24.6)	774 (25.3)	962 (31.4)	1069 (34.9)
Currently drinking alcohol, n (%)	5600 (36.6)	1404 (45.8)	1083 (35.4)	829 (27.1)	1034 (33.7)	1153 (37.6)	1139 (37.2)
Physical activity status, n (%)							
Low	5053 (33.0)	813 (26.5)	976 (31.9)	1262 (41.2)	1177 (38.4)	910 (29.7)	1017 (33.2)
Medium	5158 (33.7)	1013 (33.1)	1024 (33.4)	1069 (34.9)	1101 (35.9)	1059 (34.6)	1000 (32.6)
High	5107 (33.3)	1237 (40.4)	1063 (34.7)	732 (23.9)	786 (25.7)	1095 (35.7)	1046 (34.1)

Note: Continuous variables are presented as mean ± standard deviation or median (P25, P75), and categorical variables are presented as n (%). Abbreviations: BMI, body mass index; DBP, diastolic blood pressure; DII, dietary inflammatory index; E-DII, energy-adjusted dietary inflammatory index; Q, quintile; SBP, systolic blood pressure.

**Table 2:** Baseline daily intakes of nutrients and food groups of 15,318 Chinese adults who participated in the China Health and Nutrition Survey 1997–2015 wave based on quintiles of DII and E-DII.

Variables	ALL Participants	Quintiles of DII			*P ^a^*	Quintiles of E-DII			*P ^a^*
		Q1	Q3	Q5		Q1	Q3	Q5	
N	15,318	3063	3063	3063		3064	3064	3063	
Total energy, kcal	2213.0 ± 741.5	2845.6 ± 733.5	2215.3 ± 588.5	1593.7 ± 556.2	<0.0001	1952.2 ± 679.8	2207.8 ± 697.5	2436.0 ± 822.4	<0.0001
Carbohydrate, % E	56.0 ± 13.4	57.7 ± 13.3	55.9 ± 13.0	53.7 ± 14.0	<0.0001	54.6 ± 12.9	57.9 ± 13.3	52.8 ± 13.7	<0.0001
Protein, % E	12.3 ± 3.0	13.1 ± 3.0	12.3 ± 2.8	11.7 ± 3.1	<0.0001	14.5 ± 3.3	12.1 ± 2.4	10.5 ± 2.7	<0.0001
Fat, % E	30.0 ± 12.8	26.8 ± 12.0	30.1 ± 12.3	33.4 ± 14.1	<0.0001	28.8 ± 11.5	28.3 ± 12.5	35.2 ± 13.8	<0.0001
SFA, % E	7.0 ± 3.6	5.8 ± 3.2	7.0 ± 3.3	8.4 ± 4.2	<0.0001	6.3 ± 3.1	6.4 ± 3.2	9.1 ± 4.2	<0.0001
MUFA, % E	11.9 ± 6.0	9.9 ± 5.6	12.0 ± 5.9	14.0 ± 7.0	<0.0001	10.4 ± 5.4	11.1 ± 5.8	15.3 ± 6.7	<0.0001
PUFA, % E	7.6 ± 4.8	7.7 ± 4.5	7.6 ± 4.8	7.3 ± 5.3	<0.0001	8.4 ± 4.4	7.4 ± 4.7	7.2 ± 5.6	<0.0001
Cholesterol, mg	157.7 ± 180.2	174.4 ± 202.2	163.0 ± 185.7	134.6 ± 142.8	<0.0001	148.4 ± 170.2	145.4 ± 177.7	190.2 ± 191.0	<0.0001
Dietary fiber, g	11.7 ± 8.9	19.9 ± 12.2	10.8 ± 6.0	5.9 ± 2.8	<0.0001	16.1 ± 11.2	11.7 ± 8.6	7.5 ± 5.0	<0.0001
Vitamin A, RE	472.0 ± 786.6	639.2 ± 791.4	509.5 ± 1264.6	282.8 ± 345.6	<0.0001	625.2 ± 764.7	454.9 ± 1199.6	365.0 ± 364.2	<0.0001
Thiamine, mg	1.0 ± 0.5	1.4 ± 0.6	1.0 ± 0.3	0.6 ± 0.2	<0.0001	1.0 ± 0.5	1.0 ± 0.5	0.9 ± 0.4	<0.0001
Riboflavin, mg	0.8 ± 0.3	1.0 ± 0.4	0.8 ± 0.3	0.5 ± 0.2	<0.0001	0.9 ± 0.4	0.7 ± 0.3	0.7 ± 0.3	<0.0001
Niacin, mg	14.8 ± 6.2	19.6 ± 7.0	14.8 ± 5.0	10.2 ± 4.0	<0.0001	15.3 ± 7.1	14.7 ± 6.0	14.3 ± 5.7	<0.0001
Vitamin B_6_, μg	0.4 ± 0.2	0.6 ± 0.3	0.3 ± 0.2	0.2 ± 0.1	<0.0001	0.5 ± 0.3	0.4 ± 0.2	0.2 ± 0.2	<0.0001
Folic acid, μg	190.8 ± 92.5	275.5 ± 103.2	187.1 ± 72.0	117.0 ± 53.0	<0.0001	226.8 ± 109.1	186.9 ± 86.6	160.9 ± 71.2	<0.0001
Vitamin B_12_, g	1.6 ± 2.8	1.8 ± 3.0	1.7 ± 3.3	1.0 ± 1.4	<0.0001	1.9 ± 3.0	1.5 ± 3.3	1.3 ± 1.9	<0.0001
Vitamin C, mg	82.2 ± 69.3	126.9 ± 108.3	78.2 ± 43.0	44.6 ± 27.1	<0.0001	112.6 ± 100.7	78.7 ± 51.0	60.2 ± 39.7	<0.0001
Vitamin E, mg	31.0 ± 23.0	44.1 ± 25.2	30.4 ± 21.7	19.6 ± 17.6	<0.0001	32.8 ± 19.2	30.5 ± 20.7	30.2 ± 30.1	<0.0001
Na, mg	5618.5 ± 16,111.0	6485.7 ± 6791.6	5479.7 ± 9000.8	4454.5 ± 13,459.2	<0.0001	5197.6 ± 5020.1	5424.9 ± 8796.1	6364.3 ± 17,765.5	0.0004
K, mg	1662.8 ± 872.4	2519.7 ± 1288.5	1572.4 ± 344.3	992.6 ± 295.9	<0.0001	2080.0 ± 1283.8	1610.4 ± 590.0	1318.9 ± 470.9	<0.0001
Mg, mg	311.7 ± 140.4	466.8 ± 175.8	298.2 ± 70.7	186.7 ± 56.1	<0.0001	359.4 ± 182.5	312.0 ± 123.9	255.8 ± 91.3	<0.0001
Fe, mg	22.6 ± 11.9	33.1 ± 16.9	21.8 ± 7.7	14.3 ± 5.7	<0.0001	25.2 ± 16.5	22.3 ± 10.2	20.4 ± 9.0	<0.0001
Zn, mg	11.5 ± 4.4	15.3 ± 4.6	11.4 ± 3.3	7.7 ± 2.7	<0.0001	11.5 ± 4.8	11.5 ± 4.2	11.0 ± 4.1	<0.0001
Se, μg	41.5 ± 25.9	54.0 ± 30.9	41.7 ± 23.8	29.0 ± 14.8	<0.0001	43.7 ± 27.1	41.5 ± 27.7	38.9 ± 23.7	<0.0001
Whole grains, g	19.9 ± 58.3	40.8 ± 90.5	15.8 ± 49.4	7.1 ± 24.2	<0.0001	29.1 ± 71.3	21.4 ± 59.3	7.0 ± 45.6	<0.0001
Fruits, g	27.3 ± 72.2	42.3 ± 102.5	26.4 ± 65.0	13.1 ± 37.3	<0.0001	58.3 ± 113.0	22.8 ± 58.7	9.9 ± 34.5	<0.0001
Vegetables, g	272.7 ± 149.9	384.0 ± 185.6	270.1 ± 118.4	168.6 ± 87.7	<0.0001	342.5 ± 179.4	263.7 ± 136.4	219.2 ± 120.9	<0.0001
Nuts, g	3.2 ± 12.4	5.9 ± 18.8	3.1 ± 11.1	1.1 ± 5.7	<0.0001	5.3 ± 15.7	3.0 ± 12.2	1.5 ± 7.6	<0.0001
Legumes, g	49.3 ± 67.5	86.6 ± 85.6	47.4 ± 65.5	20.5 ± 34.1	<0.0001	77.4 ± 80.4	44.6 ± 64.3	29.2 ± 45.9	<0.0001
Dairy products, g	16.0 ± 56.1	20.1 ± 66.2	15.4 ± 55.1	11.8 ± 43.6	0.0028	30.6 ± 76.7	14.5 ± 52.8	6.8 ± 37.1	<0.0001
Eggs, g	23.6 ± 31.9	28.0 ± 35.6	23.6 ± 31.6	18.9 ± 25.3	<0.0001	30.1 ± 33.7	22.6 ± 32.0	19.3 ± 26.3	<0.0001
Fish, g	19.3 ± 34.2	23.7 ± 43.3	19.5 ± 32.7	15.3 ± 25.6	0.1372	24.0 ± 40.7	17.8 ± 31.1	17.8 ± 30.5	<0.0001
Red and processed meat, g	76.8 ± 77.0	77.7 ± 89.6	83.2 ± 79.1	66.1 ± 57.4	<0.0001	65.5 ± 69.3	70.4 ± 72.3	98.8 ± 86.1	<0.0001

Note: Continuous variables are presented as mean ± SD. Abbreviations: DII, dietary inflammatory index; E, energy; Fe, iron; Mg, magnesium; MUFA, monounsaturated fatty acid; PUFA, polyunsaturated fatty acid; Q, quintiles; RE, retinol equivalent; SFA, saturated fatty acid; Se, selenium; Zn, zinc. *^a^* Kruskal–Wallis rank-sum analysis was used to test significant differences across different quintiles of DII.

**Table 3:** Associations between DII, E-DII, and risk of all-cause mortality in 15,318 Chinese adults who participated in the China Health and Nutrition Survey 1997–2015 wave.

Variables	Quintiles					*P*-Trend	per SD	*P*
	Q1	Q2	Q3	Q4	Q5			
**DII**						
Range	(−4.08, −1.30)	(−1.30, −0.44)	(−0.44, 0.38)	(0.38, 1.30)	(1.30, 4.49)			
Median	−1.91	−0.84	−0.04	0.80	1.97			
Cases (rate, %) *^a^*	185 (6.04)	220 (7.18)	249 (8.13)	281 (9.17)	408 (13.32)			
Person year	36,138	34,544	32,944	29,688	21,172			
Model 1 *^b^*	1.00 (ref)	1.22 (1.00–1.49)	1.19 (0.98–1.44)	1.35 (1.12–1.63)	2.07 (1.72–2.50)	<0.0001	1.32 (1.24–1.40)	<0.0001
Model 2 *^c^*	1.00 (ref)	1.26 (1.03–1.54)	1.22 (0.99–1.50)	1.32 (1.06–1.64)	1.82 (1.45–2.30)	<0.0001	1.25 (1.16–1.35)	<0.0001
**E-DII**								
Range	(−4.31, −1.13)	(−1.13, −0.36)	(−0.36, 0.31)	(0.31, 1.14)	(1.14, 5.10)			
Median	−1.71	−0.72	−0.02	0.70	1.72			
Cases (rate, %) *^a^*	204 (6.66)	254 (8.29)	270 (8.81)	311 (10.15)	304 (9.92)			
Person year	28,985.46	33,236.96	33,207.36	31,980.06	27,075.31			
Model 1 *^b^*	1.00 (ref)	1.26 (1.04–1.53)	1.57 (1.25–1.96)	1.93 (1.49–2.50)	2.37 (1.76–3.19)	<0.0001	1.38 (1.25–1.52)	<0.0001
Model 2 *^c^*	1.00 (ref)	1.15 (0.94–1.39)	1.37 (1.10–1.72)	1.58 (1.22–2.06)	1.86 (1.38–2.52)	<0.0001	1.27 (1.15–1.41)	<0.0001

Note: Results are presented as HR (95% CI) estimated by using Cox proportional hazards regression models. Abbreviations: DII, dietary inflammatory index; E-DII, energy-adjusted dietary inflammatory index; Q, quintile; ref, reference. *^a^* Rate was calculated using the number of cases of death divided by the number of participants in each quintile. *^b^* Model 1 adjusted for age and sex. *^c^* Model 2 for DII adjusted for BMI, education level, region, urbanization index, physical activity, baseline hypertension, smoking, drinking, and total energy intake. Model 2 for E-DII adjusted for the same confounders except for total energy intake.

**Table 4:** Associations between DII, E-DII, and GHG emissions (gCO_2e_) in 15,318 Chinese adults who participated in the China Health and Nutrition Survey 1997–2015 wave.

Variables	Quintiles					*P*-Trend
	Q1	Q2	Q3	Q4	Q5	
**DII**						
Model 1 *^a^*	4990.16 (4882.44–5099.06)	4906.67 (4801.43–5013.07)	4894.34 (4791.00–4998.79)	4791.91 (4690.55–4894.35) *	4402.96 (4307.14–4499.84) ****	<0.0001
Model 2 *^b^*	4582.44 (4481.66–4684.35)	4838.87 (4739.72–4939.04) ****	4957.32 (4857.97–5057.67) ****	5022.64 (4920.23–5126.11) ****	4950.56 (4840.60–5061.75) ****	<0.0001
**E-DII**						
Model 1 *^a^*	4495.76 (4399.91–4592.65)	4345.75 (4250.13–4442.45)	4528.93 (4430.27–4628.68)	4979.91 (4876.66–5084.25) ****	5624.87 (5514.96–5735.86) ****	<0.0001
Model 2 *^b^*	4762.48 (4665.98–4859.96)	4706.90 (4610.81–4803.98)	4763.71 (4666.36–4862.07)	4928.21 (4828.19–5029.25) *	5218.12 (5113.71–5323.59) ****	<0.0001

Note: Results are presented as Least Squares Means (95% CL) estimated by using multiple linear regression models. Abbreviations: DII, dietary inflammatory index; E-DII, energy-adjusted dietary inflammatory index; CL, confidence level; gCO_2e_, grams of CO_2_ equivalents; GHG, greenhouse gas; Q, quintile. * Compared with Q1, *p* < 0.05; **** compared with Q1, *p* < 0.0001. *^a^* Model 1 adjusted for sex and age. *^b^* Model 2 further adjusted for BMI, region, urbanization index, educational level, physical activity, baseline hypertension, smoking status, alcohol intake, and total energy intake.

## Data Availability

The data that support the findings of this study are available from the corresponding author upon reasonable request.

## Supplementary Materials

The following supporting information can be downloaded at: https://www.mdpi.com/article/10.3390/nu17071218/s1, Table S1: Associations between DII, E-DII and risk of all-cause mortality in sensitivity analysis excluding participants those who had the cardiometabolic diseases or cancer at the baseline (N = 14,676) and who died within the first two years of follow-up (N = 15,293).
