# Supplementary material for: Associations Between Inflammatory Potential of Diet with the Risk of All-Cause Mortality and Greenhouse Gas Emissions in Chinese Adults
Source: Nutrients. 2025 Mar 30;17(7):1218. doi: 10.3390/nu17071218 (PMC11990721; doi:10.3390/nu17071218)
Supplement: Supplementary file 1 [file nutrients-17-01218-s001.zip › nutrients-3520349-supplementary.pdf]

Supplementary file

Table S1. Associations between DII, E-DII and risk of all-cause mortality in sensitivity analysis excluding those participants who had cardiometabolic diseases or cancer at the baseline (N = 14,676) and who died within the first two years of follow-up (N = 15,293).

Variables	Quintiles					*P*-trend
	Q1	Q2	Q3	Q4	Q5	
**DII**						
**Excluded participants who died within the first two years of follow-up (N = 15,293)**						
Range	(-4.08, -1.30)	(-1.30, -0.45)	(-0.44, 0.38)	(0.38, 1.30)	(1.30, 4.49)	
Median	-1.91	-0.84	-0.05	0.79	1.97	
Cases (rate, %) [a]	181 (5.92)	218 (7.13)	248 (8.11)	276 (9.03)	395 (12.91)	
Person year	36114.62	34535.82	32889.40	29655.50	21263.23	
Model 1 [b]	1.00 (ref)	1.24 (1.02-1.51)	1.22 (1.01-1.48)	1.36 (1.12-1.65)	2.07 (1.72-2.50)	<0.0001
Model 2 [c]	1.00 (ref)	1.27 (1.04-1.56)	1.24 (1.01-1.52)	1.32 (1.06-1.64)	1.77 (1.40-2.24)	<0.0001
**Excluded participants who had cardiometabolic diseases or cancer at baseline (N = 14,676)**						
Range	(-4.08, -1.30)	(-1.30, -0.43)	(-0.43, 0.37)	(0.37, 1.30)	(1.30, 4.50)	
Median	-1.91	-0.84	-0.04	0.80	1.97	
Cases (rate, %) [a]	173 (5.89)	204 (6.95)	231 (7.87)	260 (8.86)	379 (12.91)	
Person year	35153.85	33450.50	31934.73	28849.50	20860.76	
Model 1 [b]	1.00 (ref)	1.22 (1.00-1.49)	1.18 (0.97-1.44)	1.32 (1.08-1.61)	2.02 (1.66-2.44)	<0.0001
Model 2 [c]	1.00 (ref)	1.24 (1.01-1.53)	1.17 (0.95-1.46)	1.26 (1.00-1.58)	1.69 (1.33-2.16)	<0.0001
**E-DII**						
**Excluded participants who died within the first two years of follow-up (N=15,293)**						
Range	(-4.31, -1.13)	(-1.13, -0.37)	(-0.36, 0.30)	(0.31, 1.14)	(1.14, 5.10)	
Median	-1.71	-0.72	-0.02	0.70	1.72	
Cases (rate, %) [a]	199 (6.51)	252 (8.24)	263 (8.60)	307 (10.03)	297 (9.71)	
Person year	28964.77	33218.18	33214.51	31977.72	27083.39	
Model 1 [b]	1.00 (ref)	1.28 (1.05-1.55)	1.56 (1.25-1.96)	1.96 (1.50-2.56)	2.41 (1.77-3.26)	<0.0001
Model 2 [c]	1.00 (ref)	1.15 (0.94-1.40)	1.36 (1.08-1.71)	1.59 (1.21-2.08)	1.86 (1.36-2.53)	<0.0001
**Excluded participants who had cardiometabolic diseases or cancer at baseline (N=14,676)**						
Range	(-4.32, -1.13)	(-1.12, -0.36)	(-0.36, 0.31)	(0.31, 1.13)	(1.14, 5.10)	
Median	-1.70	-0.71	-0.02	0.70	1.72	
Cases (rate, %) [a]	184 (6.26)	243 (8.28)	249 (8.48)	290 (9.88)	281 (9.57)	
Person year	28598.51	32239.94	32155.59	30956.14	26299.16	
Model 1 [b]	1.00 (ref)	1.34 (1.09-1.64)	1.59 (1.26-2.01)	1.96 (1.49-2.58)	2.41 (1.76-3.31)	<0.0001
Model 2 [c]	1.00 (ref)	1.16 (0.94-1.42)	1.34 (1.06-1.70)	1.52 (1.15-2.02)	1.77 (1.28-2.44)	0.0004

Note: Results are presented as HR (95% CI) estimated by Cox proportional hazards regression models. Abbreviations: DII, dietary inflammatory index; E-DII, energy-adjusted dietary inflammatory index; Q, quintile; ref, reference.

[a] Rate was calculated using the number of cases of death divided by the number of participants in each quintile.

[b] Model 1 adjusted for age and sex.

[c] Model 2 for DII adjusted for BMI, education level, region, urbanization index, physical activity, baseline hypertension, smoking, drinking, and total energy intake for DII. Model 2 for E-DII adjusted for the same confounders except total energy intake.
